# Independent Supported Housing for Non-homeless People With Serious Mental Illness: A Pragmatic Randomized Controlled Trial

**DOI:** 10.3389/fpsyt.2021.798275

**Published:** 2022-01-21

**Authors:** Sonja Mötteli, Christine Adamus, Tim Deb, Rahel Fröbel, Jakob Siemerkus, Dirk Richter, Matthias Jäger

**Affiliations:** ^1^Department of Psychiatry, Psychotherapy and Psychosomatics, University Hospital of Psychiatry Zurich, Zurich, Switzerland; ^2^Department of Health Professions, Bern University of Applied Sciences, Bern, Switzerland; ^3^Center for Psychiatric Rehabilitation, Universitäre Psychiatrische Dienste Bern, Bern, Switzerland; ^4^University Hospital of Psychiatry and Psychotherapy, University of Bern, Bern, Switzerland; ^5^Psychiatrie Baselland, Liestal, Switzerland

**Keywords:** independent supported housing, housing rehabilitation, serious mental illness, social inclusion, mental healthcare service, evaluation, effectiveness, RCT

## Abstract

**Background:**

Independent supported housing (ISH)—services to support independent housing are recommended by current guidelines. However, there is little evidence of ISH models for non-homeless people with severe mental illness (SMI). The aim of this study was to examine the effectiveness of ISH by comparing the clinical outcomes of a newly implemented ISH service with regular housing and support services.

**Methods:**

A total of 58 adults with a broad spectrum of mental disorders experiencing housing problems were randomly assigned to either the intervention group (IG) with the possibility to use the ISH service in Zurich providing targeted, individual and flexible support for housing problems or to the control group (CG) with regular housing and support services currently available (trial registration at ClinicalTrials.gov: NCT03815604).

**Results:**

After 12 months, almost all participants of the IG were able to live independently and need for inpatient treatment could be significantly reduced. Service utilization varied between 2 and 79 h. In the CG, 70% of the participants wanted to join a waiting list for the ISH service. The results indicated that IHS was comparable to regular housing and support services in terms of social inclusion and other social and clinical outcomes such as quality of life, capabilities, needs, mental state and functioning (*p*'s > 0.05). The costs of service utilization were on average 115 Swiss Francs (about 124 USD) per participant per month.

**Conclusions:**

ISH is an effective service in housing rehabilitation in terms of social and clinical outcomes and costs. ISH is strongly preferred by service users. In line with the UN Convention on the Rights of Persons with Disabilities, access to ISH services for non-homeless people with SMI should be improved.

**Clinical Trial Registration:**

ClinicalTrials.gov, identifier: NCT03815604, December 04, 2019.

## Introduction

Adequate and stable housing conditions are well-known key components for successful psychiatric rehabilitation (1) and have become an important target in mental healthcare. As a consequence of the deinstitutionalization process, the number of people with serious mental illness (SMI) and long-term impairments requiring housing-related support increased (2). Broadly, there are three main types of support: residential care homes that provide intensive and longer-term support; supportive housing/sheltered housing with time-limited support; and independent supported housing (ISH) or floating outreach providing flexible and individual support in a permanent tenancy rented by the service users (3). To date, it is still unclear which type of support is best for an individual situation (4).

Historically, the most common approach in psychiatric rehabilitation has proposed a stepwise approach. For housing-related problems, individuals usually are admitted to a residential care home and graduate to more independent settings based on the individual's stabilization and adoption of housing skills (2). However, in practice, many service users do not move on within the expected time frame (5). In addition, current guidelines recommend practical assistance in the user's direct living environment, with the goal of fostering social inclusion in the community (6, 7). Based on the UN Convention on the Rights of Persons with Disabilities (8), service users should also have the possibility to choose the type of accommodation and support. In this sense, ISH aims to place individuals directly into an independent accommodation of the users' choice, accompanied by flexible support provided by off-site professionals for an unlimited period of time (2). There are a variety of ISH models; most of them have been designed for homeless people, such as the “Housing First” approach, which demonstrably improves housing stability (9, 10). Despite the heterogeneous conceptualization and terminology of the existing ISH models, which complicate comparisons (11), there is recent evidence that ISH is effective with respect to housing retention and stability, reducing inpatient use, and fostering social inclusion in homeless people (12–15). However, for non-homeless people, only a few observational studies are available, and these indicate mixed results (12, 14). In addition, a recent feasibility study concluded that a randomized controlled trial (RCT) with non-homeless people is not possible in this field of research because of the service users' and the staffs' distinct preferences for certain types of accommodations (4). Previous studies have already emphasized the strong preference for independent housing despite the higher risk for loneliness and isolation (16, 17). Housing satisfaction was also higher in persons with SMI living in more independent housing (18). In contrast, staff and family members tend to favor more supported and restrictive living settings (19, 20). In addition, allocation to a certain type of accommodation often seems to be influenced by its availability instead of being chosen (4). This might also be true for the larger urban areas in Switzerland, where costs of living are very high and housing is a shortage. In conclusion, there is a strong need for more evidence of the effectiveness of ISH for non-homeless people with SMI.

In this study, we compared the clinical outcomes of a newly implemented ISH service providing targeted and individual support for independent housing for people with SMI with those of regular housing and support services such as supported housing or support by social services. Specifically, we examined whether ISH and regular housing and support services will lead to similar results in terms of social inclusion as well as other social and health related outcomes such as quality of life, capabilities, social support, needs, level of mental state and functioning, and service utilization.

## Materials and Methods

This study was conducted as a pragmatic randomized controlled trial (RCT) as the method of choice for comparing medical interventions in routine care to assess real-world evidence (21, 22). The trial was conducted without blinding, with broad eligibility criteria and acceptance of the participants' treatment needs including three points of measurement (interviews at baseline, after 6, and after 12 months). Having learned from the results of a recent feasibility randomized trial (4), we intensively discussed the implementation of the RCT design with the referring health professionals and allowed freedom of choice for service utilization in both groups. The trial is part of an ongoing prospective, multi-centre cohort study in two cities in Switzerland (23).

### Setting and Intervention

In 2016, the University Hospital of Psychiatry Zurich implemented ISH as a low-threshold community-based outreach housing rehabilitation service for adults with SMI and illness-related housing problems who wish to live independently. Service users should be in psychiatric treatment (independent of the ISH service), capable of making agreements, and with a residence or intention to reside in the canton of Zurich. The main goal of ISH is to foster independent and permanent housing in a healthy and stable environment by providing psychosocial support, which aims to increase service users' social inclusion, autonomy and personal recovery. The ISH service in Zurich provides flexible, targeted and individual support as needed which, in practice, may include the support to find or keep an accommodation, facilitate contact with landlords, the social environment or mental health services and the provision of housing skills. These services are provided without time limitations, up to four h a week by non-medical staff with nursing and social work education. The team is completed by psychiatrists, who can be consulted if needed.

### Participants

**Figure 1:**
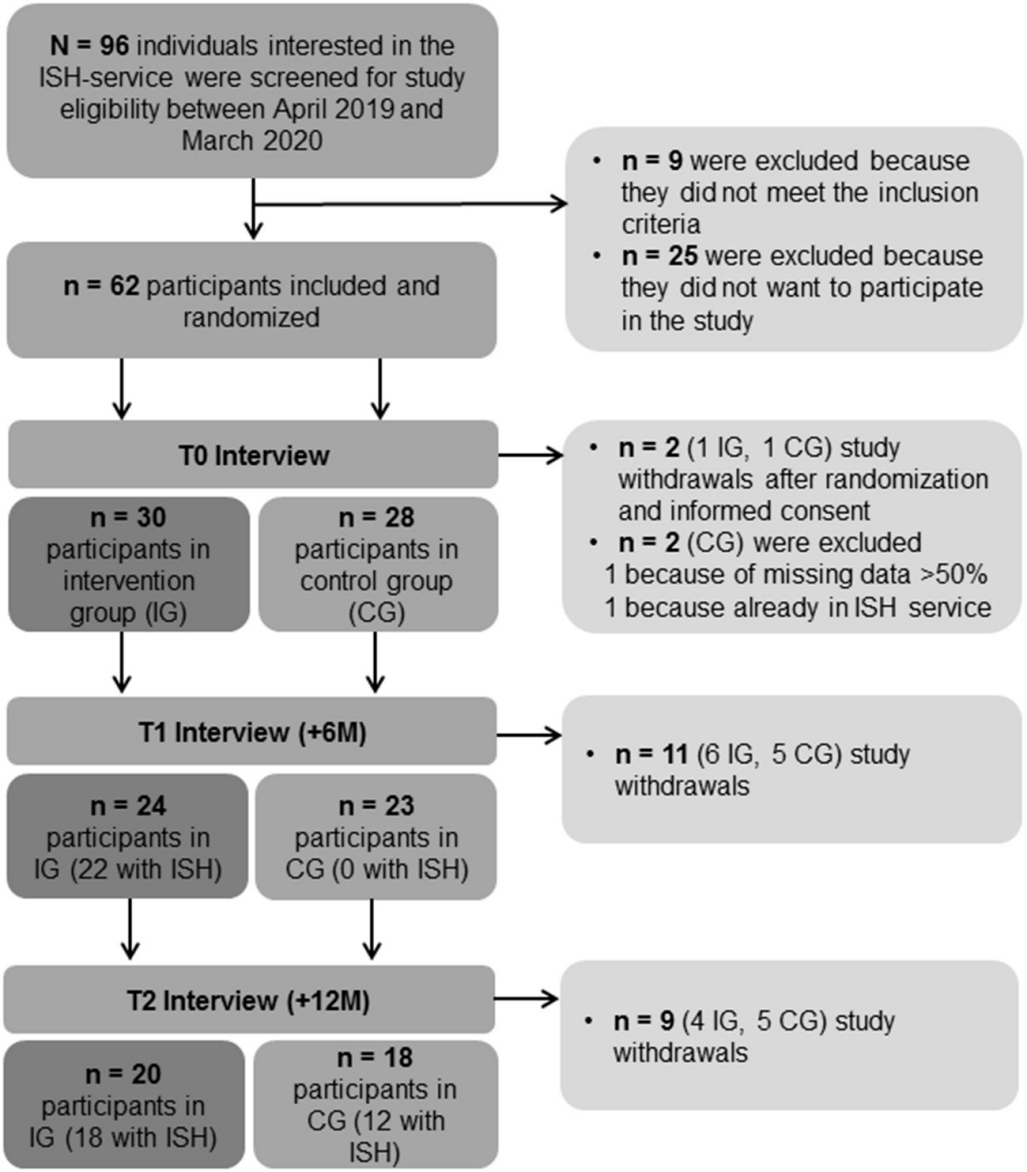
Flowchart of the participants' inclusion process.

Between April 2019 and March 2020, we screened all individuals who were interested in the ISH service in Zurich for study eligibility (see Figure 1). These were adults with a psychiatric disorder and heterogeneous housing problems who wished to live independently or to remain independent. Almost all of them were referred by social workers or psychiatrists affiliated with the University Hospital of Psychiatry Zurich or other psychiatric and social institutions located in the Canton of Zurich. During the recruitment period, access to the ISH service was limited to study participants only, which was possible due to the scarcity of comparable services in Zurich. Inclusion criteria were a mental disorder according to the International Classification of Diseases, 10th edition (ICD-10) and related housing problems, age between 18 and 65 years, the ability to communicate in German and, if indicated, to take prescribed medication, having a source of income including social welfare to pay for housing, and the ability to give written informed consent. Exclusion criteria were severe cognitive impairments, intoxication, delirium, dementia, mental incapacity, acute risk of self-harm or harm to others. During a period of one year, 62 individuals of a total of 96 screened patients could be successfully included in the study (see Figure 1) based on the calculated sample size of 28 participants needed for each group [for details see (23)].

### Procedure and Study Conditions

The study coordinator explained the purpose and procedures of the study to all individuals who were interested in the ISH service. If the participants gave written informed consent, they were randomly assigned to either the intervention group (IG) or the control group (CG). For each participant, the result of randomization was enclosed in an envelope based on a randomization sheet, which had been calculated before the start of the study with block-randomization using the statistical software R. The randomization sheet was not accessible to the study coordinator to prevent the possibility of spontaneous modifications. After the group assignment, the participants completed the baseline interview (T0). The participants in the IG were invited to start the ISH service within four weeks of the baseline interview. The participants of the CG (*n* = 22 participants who were already living in an independent accommodation, *n* = 1 participant in a shared apartment, and *n* = 1 homeless participant, see Table 1) could not use the ISH service and were referred back to the support service they were already receiving. This means that for the CG, a social worker or clinical therapist was the only person in charge of housing support along with all other responsibilities. In most of the cases, housing problems were related to the need for finding a more appropriate housing form, for instance, due to a precarious housing situation or pending termination of the rental agreement. Four participants of the CG were cared for in residential care homes over the course of the study. Although the CG included diverse support forms, all of these services did not include an established ISH service. In addition, the participants of the CG and their referring therapists received a list of various established standard services in housing rehabilitation, such as residential care homes, shared or non-shared apartments offered by residential agencies, host families, and social services. Participants of the CG were also free to seek help at other support services such as communal services or friends and family. However, at T1 (+6 months), due to ethical reasons with regard to the scarcity of comparable services in the Canton of Zurich, the participants in the CG could join a waiting list for the ISH service. Six months (T1) and 12 months (T2) after the baseline interview (T0), the participants were asked to take part in the follow-up interviews. These were conducted by trained assessors, lasted about 1.5 h, and took place at the University Hospital of Psychiatry Zurich or a place of the participants' choice. Since the emergence of the Covid-19 pandemic, interviews were also conducted by telephone at the request of the participants. In addition, the participants received ten Swiss Francs for each completed interview to minimize potential dropouts in the CG.

**Table 1:** Participants' characteristics at baseline T0.

	**IG (*****n*** **=** **30)**		**CG (*****n*** **=** **28)**	
	***n* (%) or M**	**SD**	***n* (%) or M**	**SD**
Gender, male	10 (33.3)		12 (42.9)	
Age	40.43	12.25	44.36	9.64
Swiss nationality	19 (63.3)		17 (60.7)	
Education				
No education	2 (6.7)		2 (7.4)	
Compulsory schooling	8 (26.7)		10 (37.0)	
Vocational education	11 (36.7)		6 (22.2)	
Matura	5 (16.7)		4 (14.8)	
Higher vocational education	2 (6.7)		0	
University	2 (6.7)		5 (18.5)	
Source of income				
Salary	3 (10.0)		3 (10.7)	
Pension	15 (50.0)		8 (28.6)	
Social-welfare benefits	9 (30.0)		13 (46.4)	
Support by family	2 (6.7)		1 (3.6)	
Other source	1 (3.3)		3 (10.7)	
Main psychiatric diagnosis (ICD-10)				
F1	5 (16.7)		1 (3.6)	
F2	9 (30.0)		9 (32.1)	
F3	11 (36.7)		11 (39.3)	
F4	2 (6.7)		5 (17.9)	
F6	3 (10.0)		2 (7.1)	
Housing situation				
Independent accommodation	21 (70.0)		22 (78.6)	
Residential care home	3 (10.0)		4 (14.3)	
Shared apartment	2 (6.7)		1 (3.6)	
Host family	2 (6.7)		0	
Homelessness	2 (6.7)		1 (3.6)	

*IC, intervention group; CG, control group; one person did not indicate the education level*.

### Measures

The interviews included self-assessment questionnaires that addressed social inclusion, quality of life, capabilities, social support, needs, mental state, utilization of mental healthcare as well as external assessment questionnaires covering functioning, ISH utilization and costs.

Measures are described in detail below including information on central tendency and dispersion of all participants at baseline T0.

*Social inclusion* was assessed using the validated German version of the Social Functioning Scale (SFS) including seven subscales (24, 25). Most of the 73 items could be answered on a 4-point Likert scale with higher mean scores indicating better social inclusion. Raw scores were translated into standardized scale scores with *M* = 100 and SD = 15 (*M* = 105.0, SD = 8.5, range = 82.1–121.4, Cronbach's alpha = 0.82, *n* = 58).

*Quality of life* was evaluated via the Manchester Short Assessment of Quality of Life (MANSA) (26) using a German version translated by Röpcke, B. and Linau, N. (2000, unpublished). Most of the 16 different life domains were measured on a 7-point Likert scale (1–7) with higher mean scores indicating better quality of life (*M* = 3.7, SD = 0.9, range = 1.7–5.8, Cronbach's alpha = 0.72, *n* = 58).

*Capabilites* were assessed using the validated German version of the Oxford Capabilities Questionnaire—Mental Health (OxCAP-MH) encompassing 16 items on a 5-point Likert scale (27, 28). Raw scores were translated into standardized scale scores ranging from 0 to 100 with higher scores indicating better capabilities (*M* = 59.2, SD = 15.8, range = 25.0–95.3, Cronbach's alpha = 0.80, *n* = 58).

*Social support* was measured using the validated German adaption of the ENRICHED Social Support Inventory (ESSI-D) including 5 items on a 5-point Likert scale (1–5) with higher sum scores indicating better social support (29, 30) (*M* = 17.1, SD = 5.3, range = 5.0–25.0, Cronbach's alpha = 0.85, *n* = 58).

*Needs* were assessed using the validated German version of the Camberwell Assessment of Need—Short Appraisal Schedule (CANSAS) including 22 items of different domains regarding health and functioning (31, 32). Needs (met needs, unmet needs) were summed up with higher sum scores indicating more needs (*M* = 7.3, SD = 2.8, range = 1.0–14.0, Kuder-Richardson-20 score = 0.59, *n* = 58).

*Mental state and functioning* of the participants were measured by three different scales. For assessing mental state, the 9-item Symptom Checklist (SCL-K-9) (33, 34) was completed by the participants. A 5-point Likert scale (0–4) was used, with lower mean scores indicating a better mental state (*M* = 1.6, SD = 0.9, range = 0.1–3.4, Cronbachs' alpha = 0.85, *n* = 58).

The Health of the Nation Outcome Scales (HoNOS) and the modified Global Assessment of Functioning Scale (m-GAF) were completed by an ISH staff member (IG) or by the study interviewer (CG). In the IG at baseline (T0), *n* = 5 ratings could not be completed. The validated German version of the HoNOS included 12 items on a 5-point Likert scale (35, 36) (*M* = 1.1, SD = 0.4, range = 0.3–2.2, Cronbach's alpha = 0.50, *n* = 53). The m-GAF was rated on a scale in the range 0–100, with higher scores indicating better functioning (37).

*Service utilization* of mental healthcare was assessed using the German adaption of the Client Sociodemographic and Service Receipt Inventory (CSSRI-EU) which included different areas, such as utilization of healthcare services, intake of psychotropic medication and contact with criminal justice services (38).

*Service utilization and costs* of the ISH service were assessed using the Swiss medical tariff reimbursement tool for outpatient services (TARMED). The costs were calculated based on the number and duration of contact with the ISH service (TARMED codes).

### Data Analysis

We analyzed data by an intention-to-treat approach comparing the means of all outcome variables using *t*-tests for independent samples between the IG and CG for all three measurement points based on a 5% significance level. Scale values (means or sum scores) were calculated if at least 66% of the items were completed. The missing data (<3.5% of data points) were replaced by scale means. McNemar's tests were used for data analyses of service utilization within one treatment group. Statistical analyses were performed using IBM SPSS (version 26 for Windows, IBM Corp., 2019).

## Results

### Sample Characteristics and Outcome of Randomization

Of the 58 included participants (see Figure 1), 42 were referred by clinicians of the University Hospital of Psychiatry Zurich, 11 were referred by therapists and social workers of other institutions, and five were self-referrals. Most of the participants had an affective or psychotic disorder, while 57% reported the first occurrence of psychiatric problems below or similar to the age of 25 years; 74% reported having one or more additional somatic diagnoses. As shown in Table 1 more than 70% of the participants lived independently in an accommodation. The majority of the participants (76%) had no prior experience with residential care homes, and only seven participants needed a guardianship for financial issues. Reasons for study participation were, among others, needing support to find or keep an accommodation, practical assistance for flat clearances, and troubles with landlords or neighbors. Table 1 shows that the participants' characteristics were similarly distributed between the IG and the CG (non-significant). Baseline characteristics showed also no significant differences at T1 and T2.

### Participants' Preference for Independent Housing

Within four weeks after the randomization, the participants of the IG were invited to start the ISH service. Of these 30 participants, 26 used the ISH service whereas 4 participants decided not to use it for various reasons, such as unexpected changes in housing conditions. Participants of the CG (*n* = 28) could not use the ISH service. The majority of the participants lived independently and received at least support from their therapists and social workers. After six months (T1), 16 participants (70%) in the CG reported continued interest in the ISH service. They were put on a waiting list and were later invited to start the ISH service after the second interview, depending on the capacity of the ISH team, on average M_d_ = 281.5 days after T1. Therefore, after 12 months (T2), utilization of the ISH service (hours) in the CG was still low.

### Comparison of Clinical Outcomes Between Intervention and Control Groups

During the observed study period of one year (T0–T2), 24 participants changed their housing situation (12 in IG and 12 in CG). In the IG, four participants moved from supported housing (including residential care homes, shared apartments, host families) to independent housing (apartments rented by the participants). Only one person—who did not use the ISH service—moved from independent housing to supported housing. Contrastingly, in the CG, three participants moved from independent housing to supported housing, one person became homeless, and one person moved into another residential care home. The remaining 14 participants of both groups independently moved from one apartment to another. There were no differences in housing satisfaction between the IG and the CG. During the entire study period, the self-reported service utilization of psychiatric and somatic treatments was high in both groups (Table 2). Participants who used the ISH service more frequently reported involvement in psychiatric treatment. A reason for this difference could be that this was a precondition to receive the service. Given that many participants were recruited during inpatient treatment it was not surprising that the absolute number of participants receiving inpatient treatment decreased from T0 to T2. However, in the IG, the decrease in inpatient treatment was significant (*p* = 0.004) whereas in the CG, it was not significant (*p* = 180).

**Table 2:** Self-reported service utilization of the participants.

**Service utilization during**	***n* (%)**	***n* (%)**	χ^2^*	***p***
**last 6 months**				
	**Baseline T0**			
	**IG (*n* = 30)**	**CG (*n* = 28)**		
Psychiatric treatment	30 (100.0)	27 (96.4)	1.09	0.296
Inpatient treatment	16 (53.3)	10 (35.7)		
Psychotropic medication	28 (93.3)	17 (60.7)	8.86	0.003
Somatic treatment	15 (50.0)	20 (74.1)	3.48	0.062
Inpatient treatment	2 (6.7)	4 (14.3)		
Contact with criminal justice	6 (20.0)	10 (35.7)	1.79	0.181
	**T1 (T0** **+** **6M)**			
	**IG (*****n*** **=** **24**	**CG (*****n*** **=** **23)**		
Psychiatric treatment	24 (100)	19 (82.6)	4.56	0.033
Inpatient treatment	8 (33.3)	4 (17.4)		
Psychotropic medication	20 (83.3)	15 (65.2)	2.03	0.154
Somatic treatment	18 (75.0)	11 (47.8)	3.67	0.055
Inpatient treatment	3 (12.5)	1 (4.3)		
Contact with criminal justice system	1 (4.2)	5 (21.7)		0.097
	**T2 (T0** **+** **12M)**			
	**IG (*****n*** **=** **20)**	**CG (*****n*** **=** **18)**		
Psychiatric treatment	19 (95.0)	18 (100.0)	0.924	0.336
Inpatient treatment	1 (5.0)	4 (22.2)		
Psychotropic medication	18 (90.0)	13 (72.2)	1.992	0.158
Somatic treatment	13 (65.0)	11 (61.1)	0.062	0.804
Inpatient treatment	1 (5.0)	0		
Contact with criminal justice system	3 (15.0)	6 (33.3)		0.260

*chi-squared test or Fisher's exact test if n < 5; p < 0.05, two-sided.

At baseline (T0), scale scores showed slightly better values for the CG with significant differences between the IG and the CG for capabilities and a statistical trend for quality of life (see Table 3). After 6 months (T1), there were no significant differences between the IG and CG except for capabilities and a statistical trend for quality of life in line with the baseline values. After 12 months (T2), and contrary to T1, most scale scores showed greater improvement in the IG compared to the CG, with no significant differences between the two conditions. Details of the scales are shown in Table 3.

**Table 3:** Differences in social inclusion, capabilities, quality of life, social support, needs, mental state and functioning between the intervention group (IG) and the control group (CG) at baseline, T1 and T2.

**Scales**	***M***	**SD**	***M***	**SD**	**95% CI**		***p***
	**Baseline T0**						
	**IG (*****n*** **=** **30)**		**CG (*****n*** **=** **28)**				
SFS	104.59	8.59	105.38	8.62	−5.32	3.73	0.727
OxCAP-MH	55.23	16.85	63.40	13.58	−16.26	−0.09	0.048
MANSA	3.51	0.98	3.90	0.71	−0.85	0.06	0.088
ESSI-D	16.74	5.55	17.58	4.97	−3.62	1.94	0.548
CANSAS	6.97	2.91	7.57	2.77	−2.10	0.89	0.421
SCL-K-9	1.64	0.97	1.57	0.83	−0.41	0.55	0.770
HoNOS	1.16	0.40	1.12	0.44	−0.20	0.27	0.762
m-GAF	51.84	12.58	52.50	9.49	−6.76	5.44	0.829
	**T1 (T0** **+** **6M)**						
	**IG (*****n*** **=** **24)**		**CG (*****n*** **=** **23)**				
SFS	103.96	8.04	106.22	7.95	−6.96	2.44	0.338
OxCAP-MH	56.95	12.80	67.97	14.69	−19.10	−2.93	0.009
MANSA	3.72	1.07	4.31	1.04	−1.21	0.03	0.062
ESSI-D	16.58	4.76	16.72	6.39	−3.44	3.16	0.932
CANSAS	6.83	3.13	6.78	3.74	−1.97	2.07	0.960
SCL-K-9	1.66	0.94	1.32	0.84	−0.19	0.86	0.206
HoNOS	1.00	0.39	0.80	0.42	−0.04	0.44	0.094
m-GAF	55.92	12.33	58.65	11.30	−9.69	4.22	0.433
	**T2 (T0** **+** **12M)**						
	**IG (*****n*** **=** **20)**		**CG (*****n*** **=** **18)**				
SFS	102.66	11.11	99.05	8.18	−2.87	10.09	0.266
OxCAP-MH	57.44	15.08	56.83	17.07	−9.97	11.18	0.908
MANSA	3.92	0.93	3.80	1.01	−0.53	0.76	0.718
ESSI-D	15.84	4.75	16.56	5.39	−4.06	2.62	0.665
CANSAS	5.60	2.04	7.22	3.15	−3.35	0.11	0.065
SCL-K-9	1.44	0.92	1.62	0.80	−0.75	0.40	0.536
HoNOS	1.03	0.39	1.30	0.47	−0.55	0.01	0.061
m-GAF	55.70	12.46	53.89	12.01	−6.26	9.88	0.653

*SFS, Social Functioning Scale; OxCAP-MH, Oxford Capabilities Questionnaire – Mental Health; MANSA, Manchester Short Assessment of Quality of Life; ESSI-D, ENRICHED Social Support Inventory; CANSAS, Camberwell Assessment of Need – short Appraisal Schedule; SCL-K-9, 9-item Symptom Checklist; HoNOS, Health of the Nation Outcome Scales; m-GAF, modified Global Assessment of Functioning Scale*.

### Utilization of the ISH Service and Costs

Of the 26 participants who used the ISH service in the IG, 11 discontinued the support during the study period of 12 months, while 15 participants continued using it. ISH provided between 2.3 and 78.8 h (M_d_ = 11.1 h) of support during 30–365 days (M_d_ = 318 days). This corresponds on average to 15 min per participant per week. Participants with higher HoNOS (r_s_ = 0.34, *p* = 0.042) and lower GAF scores (r_s_ = 0.43, *p* = 0.008) at baseline needed more support. The service utilization resulted in M_d_ = 1,202 Swiss Francs (about 1,291 USD), meaning an average cost of 115 Swiss Francs per participant per month. For individuals who were living in Zurich in 2021 and were dependent on social-welfare benefits, monthly costs for independent supported housing were about 2,321 Swiss Francs per month (1,006 Swiss Francs for basic needs, 1,200 Swiss Francs for rent costs).

## Discussion

To the best of our knowledge, this is the first RCT to evaluate the effectiveness of the ISH approach for non-homeless people with SMI in comparison to a control group of regular housing and support services. Our results indicate that ISH is a cost-effective service in housing rehabilitation. In addition, ISH is strongly preferred by service users.

Participants' recruitment and randomization into either the IG with ISH service or the CG with regular housing and support services could be finalized within one year with the result of balanced groups in terms of the participants' sociodemographic and illness-related characteristics. In contrast, a previous study had to conclude that randomization into either ISH or supported housing was not feasible (in Great Britain) (4). However, in the present study, participants in the CG could use different standard services and, after six months, they had the option to join a waiting list if they were still in need of ISH. These options, in combination with the scarcity of the examined service, decisively enhanced the acceptance of the RCT among the participants and their referring healthcare professionals. At the same time, the option to join a waiting list hindered the conduction of a long-term study over two years as was originally planned (23). After six months, 70% of the participants in the CG were still interested in the ISH service. Considering the participants' poor mental health and the seriousness of their housing problems, the long wait clearly revealed the participants' preferences. Previous findings have already shown that people with SMI strongly prefer to live independently (4, 16, 17).

Furthermore, this study contributes to findings regarding the effectiveness of ISH for non-homeless people with SMI, which have provided only inconsistent results (11, 13, 14). After one year, all participants of the IC—with the exception of one person who did not used the ISH service—were able to live independently. In contrast, in the CG, 4 participants had to give up independent housing. These results indicate that people with SMI who live independently and experience housing problems seem to be at risk to loose independent housing without ISH support. The need for inpatient treatment decreased in both groups from T0 to T2, however, with a significantly larger proportion in the IG. Levels of social inclusion in the IG were comparable to those of the CG. However, contrary to expectations, levels of social inclusion and levels of social support declined during the observed study period in both groups. Probably this decline is due to the Covid-19 pandemic, which emerged during the study period. Regarding the measures of capabilities, quality of life and needs, there was a trend toward better scale scores in the IG compared to decreased scale scores in the CG over the three measurements. Additionally, self-ratings and external assessments showed similar levels of the participants' severity of symptoms and functionality in both groups. These results may indicate the effectiveness of the examined ISH service, which aimed to foster independent and permanent housing in a healthy and stable environment through very targeted and individualized services such as the support to find or keep an accommodation and the provision of housing skills. In addition to the clinical outcomes, ISH with costs of about 2,321 Swiss Francs per month seems to be cost-effective in comparison to more institutionalized support forms. In 2021, the average monthly cost of typical residential care homes in the Canton of Zurich was around 4,500 Swiss Francs (including psychosocial care and food). However, due to the Covid-19 pandemic, the mean utilized hours might be underestimated because some outreach services were omitted or replaced by telephone appointments.

We examined only one specific ISH service in a distinct setting in Switzerland, which limits the generalizability of the results. Given that the CG could benefit from a rather high availability of social services in the Canton of Zurich and that regular housing support such as residential care homes are easily available, our results all the more underline the relevance of ISH services.

As a limitation of this study, the clinical outcomes cannot be analyzed and interpreted in more detail due to a number of reasons. First, there were many study withdrawals. A substantial proportion of the participants who successfully terminated the ISH service (33% in the IG) or used a suitable alternative (36% in the CG) did not continue to participate in our study. Second, the high preference for ISH hindered the evaluation of the comparative long-term effectiveness of the ISH approach. Third, we did not further examined the various support forms in the CG. Pragmatic trials would require larger sample sizes and longer observation periods to detect effects (21). In line with a previous feasibility study (4), we suggest that further studies in housing rehabilitation may use more user-friendly and innovative research designs, such as observational studies, including statistical techniques to control for confounding.

In conclusion, the results of this RCT indicate that ISH is an effective service in housing rehabilitation in terms of social inclusion, other social and clinical outcomes and costs. ISH enables the users to live independently and could reduce the need for inpatient treatment through very targeted and individualized services such as the support to find or keep an accommodation and the provision of housing skills. ISH is strongly preferred by service users and also suggested by current guidelines. Based on the UN Convention on the Rights of Persons with Disabilities (8), which demands freedom of choice for the type of support, access to ISH services for non-homeless people with SMI should be improved.

## Data Availability Statement

The dataset is available from the corresponding author after written agreement with the principal investigators.

## Ethics Statement

The studies involving human participants were reviewed and approved by the Swiss Association of Research Ethic Committees (swissethics), Reference No. 2018-02381. The patients/participants provided their written informed consent to participate in this study.

## Author Contributions

DR is the sponsor-investigator of the current study and DR and MJ are the principal investigators. The study was conducted by SM, TD, and CA. RF and JS also participated in the implementation of the study design. All authors contributed to the article and approved the submitted version.

## Funding

This research was funded by the Swiss National Science Foundation SNF (10531C_179451) after being peer-reviewed. The SNF had no role in the design and conduct of the study, data collection, analysis or interpretation of the data, preparation, review and approval of the manuscript.

## Conflict of Interest

The authors declare that the research was conducted in the absence of any commercial or financial relationships that could be construed as a potential conflict of interest.

## Publisher's Note

All claims expressed in this article are solely those of the authors and do not necessarily represent those of their affiliated organizations, or those of the publisher, the editors and the reviewers. Any product that may be evaluated in this article, or claim that may be made by its manufacturer, is not guaranteed or endorsed by the publisher.

## Abbreviations

ISH: independent supported housing
RCT: randomized controlled trial
SMI: severe mental illness.
